# Computational Selection of Transcriptomics Experiments Improves Guilt-by-Association Analyses

**DOI:** 10.1371/journal.pone.0039681

**Published:** 2012-08-07

**Authors:** Prajwal Bhat, Haixuan Yang, László Bögre, Alessandra Devoto, Alberto Paccanaro

**Affiliations:** 1 School of Biological Sciences, Royal Holloway University of London, Egham, United Kingdom; 2 Department of Computer Science, Royal Holloway University of London, Egham, United Kingdom; 3 Centre for Systems and Synthetic Biology, Royal Holloway University of London, Egham, United Kingdom; University of Toronto, Canada

## Abstract

The Guilt-by-Association (GBA) principle, according to which genes with similar expression profiles are functionally associated, is widely applied for functional analyses using large heterogeneous collections of transcriptomics data. However, the use of such large collections could hamper GBA functional analysis for genes whose expression is condition specific. In these cases a smaller set of condition related experiments should instead be used, but identifying such functionally relevant experiments from large collections based on literature knowledge alone is an impractical task. We begin this paper by analyzing, both from a mathematical and a biological point of view, why only condition specific experiments should be used in GBA functional analysis. We are able to show that this phenomenon is independent of the functional categorization scheme and of the organisms being analyzed. We then present a semi-supervised algorithm that can select functionally relevant experiments from large collections of transcriptomics experiments. Our algorithm is able to select experiments relevant to a given GO term, MIPS FunCat term or even KEGG pathways. We extensively test our algorithm on large dataset collections for yeast and Arabidopsis. We demonstrate that: using the selected experiments there is a statistically significant improvement in correlation between genes in the functional category of interest; the selected experiments improve GBA-based gene function prediction; the effectiveness of the selected experiments increases with annotation specificity; our algorithm can be successfully applied to GBA-based pathway reconstruction. Importantly, the set of experiments selected by the algorithm reflects the existing literature knowledge about the experiments. [A MATLAB implementation of the algorithm and all the data used in this paper can be downloaded from the paper website: http://www.paccanarolab.org/papers/CorrGene/].

## Introduction

In the past decade, efforts for elucidating gene function have gained new impetus with the emergence of large scale transcriptomics and protein-protein interaction experiments. These datasets are mined to identify groups of genes sharing similar features, which implies that they may share similar functions – this principle has often been called Guilt-By-Association (GBA) [1]–[4]. Amongst the various high-throughput data types available, transcriptional profiling is currently the most abundant. Relying on the concept of GBA, numerous strategies have been developed to extract functional information from transcriptomics data including clustering-based techniques and co-expression network analyses. GBA-based analyses often begin with the calculation of similarity between gene expression profiles using a metric such as Pearson's correlation. Often, this has been performed over large heterogeneous collections of experiments. One reason behind this approach is that correlating gene profiles over a larger number of experiments would result in more robust correlations as weak expression signatures are combined over many datasets. In fact, the significance of the correlation between vectors is likely to increase with the size of the vectors.

The analysis of large collections of microarray datasets has been useful to reveal the transcriptional responses of genes expressed similarly through a range of experimental conditions [5]. However, it may not be optimal for revealing the function of genes whose expression is condition-specific [6] and some authors have suggested that in these cases a smaller set of condition-related experiments should instead be used [7].

**Figure 1:**
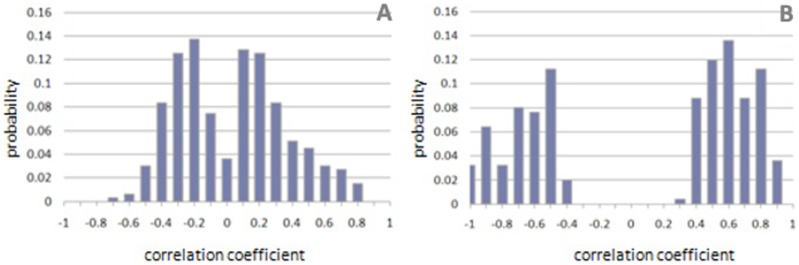
Correlation coefficients between genes in a given GO category depend on the experiments being used. Distribution of correlation coefficients for genes in the GO category GO:009753 “Response to Jasmonic Acid stimulus” calculated using (A) an heterogeneous collection of 44 experiments and (B) a manually selected set of two experiments, which were deemed to be functionally relevant to jasmonic acid response based upon literature knowledge. Only statistically significant correlation coefficients (p-value<0.05) were considered in order to account for the different vector lengths.

To set the stage for our work, we analyze this fact by looking at the distribution of the statistically significant correlation coefficients for the genes in the GO Biological Process “Response to Jasmonic acid stimulus” in the model plant *Arabidopsis thaliana*. Figure 1 compares the distribution obtained using a large heterogeneous collection of 44 experiments (756 microarrays) (Fig. 1a) with the distribution obtained using a smaller set of 2 experiments (24 microarrays) which were manually selected according to literature knowledge relevant to “Response to Jasmonic acid” (Fig. 1b) – experimental details are given in the Methods section.

We observe that the two distributions are strikingly different: when using all the experiments, only 18.6% of the gene pairs show an absolute correlation above 0.5, while using only the selected experiments, 81.1% of the gene pairs have an absolute correlation above 0.5. The inclusion of all the experiments in the calculation of correlation leads to significantly lower correlations overall, thus limiting the effectiveness of GBA. In fact, if the correlation among genes in a functional category is close to zero, the genes that may belong to that category cannot be inferred based on correlation with genes already annotated to that category. Importantly, we obtained similar results using most GO categories, across different functional classification systems (e.g. MIPS) and across organisms.

In a given experiment, one possible reason for low correlation among genes in the same functional category could be that the functional process to which these genes belong has not been activated under those experimental conditions. Thus, in the absence of signal, we would just be correlating experimental noise. In fact, if two genes belong to a process which has been activated in the experiments, they would likely be highly correlated in spite of the (unavoidable) experimental noise. However, if the process had not been activated the experimental noise would still be recorded, thus resulting in poor correlation between the genes. We analyzed this phenomena in depth using artificial data – see Supplementary Information **S1**.

Poor correlation could also result from biological phenomena such as cross-talk in the regulatory pathways. When two genes belong to the same pathway, but one of them also functions in a different one, the two genes can be seen as highly or poorly correlated depending on the pathways being activated. One such example would be phytochrome and cryptochrome-mediated signalling in *Arabidopsis thaliana* where the effects exerted by different proportion of blue, red or far red light in white light is dependent upon the condition used [8]. In experiments studying hypocotyl elongation, under short exposures to blue light in a red light background, the activity of the *CRY1* gene and *PHYB* gene is found to be correlated. However, during prolonged exposure to blue light, the activity of *CRY1* and *PHYB* are seen to be independent. Therefore, although *CRY1* and *PHYB* participate in the same biological process, any correlation between them would be condition-specific.

Importantly, such phenomena could have a profound effect on functional analyses as the correlation signal dilutes quickly. Therefore, when analysing large collections of microarrays, the inclusion of datasets in which genes appear not to be correlated, will result in low overall correlations even for genes that belong to the same biological process. We performed an in-depth analysis of the rate at which correlation dilutes using artificial data – see Supplementary Information **S2**.

The remarkable difference between Figure 1A and Figure 1B reflects the findings of Adler et al. [7] that acknowledged the pitfalls of using large microarray collections in co-expression analyses and suggested selecting the relevant datasets based on literature knowledge. However, identifying experiments based on literature knowledge alone is a non-trivial task as the literature knowledge relevant to a functional category of interest is seldom exhaustive. Further, the relevance of an experiment to a certain biological process may not be immediately obvious and experiments which are deemed irrelevant by a researcher could in fact withhold significant information regarding the biological process of interest as well as cross-talk between pathways.

In this paper, we present a novel algorithm for systematically selecting from large collections those experiments which are relevant to a given functional category or pathway. Importantly, the algorithm is able to identify relevant experiments not obvious by searching the literature on the experiment. Our results show that using experiments selected by the algorithm leads to substantially improved correlation between genes in the same functional category compared to using large heterogeneous collections of experiments. As a consequence, we also demonstrate that using correlation obtained with the selected experiments leads to substantial improvements in GBA-based function prediction independently of the species and of the functional classification schemes adopted. Finally, we show how our algorithm can improve GBA-based pathway reconstruction.

It is important to note here that the fundamental ideas behind our algorithm are not specific to microarray data. Emerging gene expression measurement technologies such as RNA-seq will eventually lead to the availability of large collections of data. Our algorithm can equally well be used to select experiments from large RNA-seq experiment collections.

## Results

Given a functional category of interest such as GO Biological Process term or a biochemical pathway and a set of microarray experiments our task is to select a subset of experiments that is optimal at differentiating the genes in that functional category from the remaining ones – which we shall call *background* genes. Since the chosen subset of experiments should be constituted by experiments that most perturb the genes in the functional category of interest, we shall refer to these experiments as the *relevant* experiments.

Our idea is to choose a feature that, if an experiment is relevant, would be able to discriminate between the genes in the category of interest and the background genes. The set of relevant experiments can then be found by maximizing the discriminatory ability of such a feature. The feature we chose is Pearson's correlation coefficient and we used a *t*-test to measure its discriminatory ability – whether the mean of the correlation coefficients for the genes of interest is significantly higher than that of the background.

**Figure 2:**
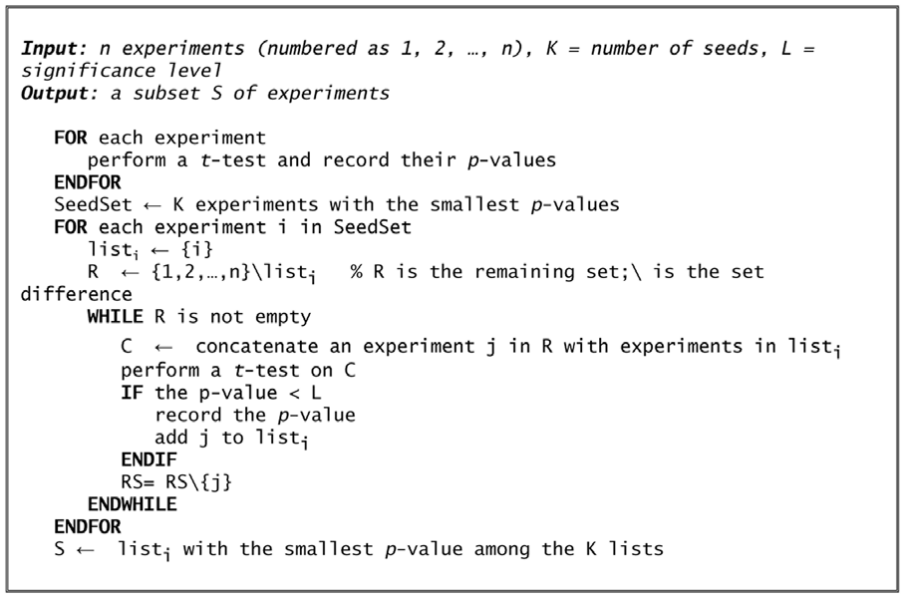
Pseudo-code of the experiment selection algorithm. The *t*-tests are performed between two sets of correlations obtained from two classes of gene pairs: gene pairs where both genes belong to the functional category of interest (panel A in Figure 3); and gene pairs where one gene belongs to the functional category of interest and the other one to the background (panel B in Figure 3).

Clearly, an exhaustive search of the space of possible subsets of experiments is computationally intractable for large microarray datasets (the number of possible subsets of a set of *n* experiments is *2^n^)*. Therefore, a ‘brute force’ approach that analyzes every possible combination of experiments would not be feasible for typical microarray collections containing a large number of experiments. For example, the set of 44 Arabidopsis microarray experiments that we have used in this work would require analyzing over 17,000 billion combinations. Therefore, this paper presents an efficient greedy heuristic which was able to select a set of experiments with high discriminatory ability while retaining a quadratic complexity. Here we give an informal description of the procedure while the pseudo-code of the algorithm is presented in Figure 2.

**Figure 3:**
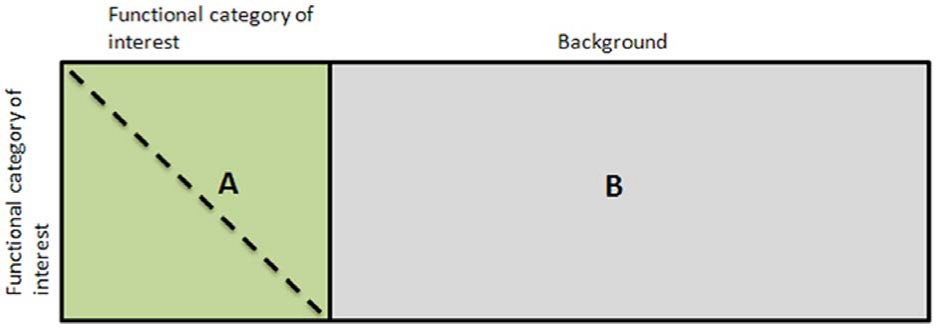
Correlation matrices used in the algorithm. A graphical depiction of the correlation matrix for gene pairs where both genes belong to the functional category of interest (A) and the correlation matrix for gene pairs where one gene belongs to the functional category of interest and the other one to the background (B). Note that elements on the main diagonal of A are equal to one, and that A is symmetric (as indicated by the dashed line). *t*-tests in our algorithm are calculated between the sets of correlations in A and B (for A we use the upper triangular part only).

Our analysis assumes that we are given a certain functional category and a set of *n* microarray experiments, each comprising several time-points or conditions. The procedure begins by performing a *t*-test for every experiment in the microarray collection assessing whether the correlation between gene pairs where both genes in the pair belong to the functional category of interest (denoted by A in Figure 3) is greater than correlation between gene pairs where only one gene belongs to the functional category of interest (denoted by B in Figure 3). Note that we do not consider correlations where neither genes in the pair belong to the functional category (the rationale for this is discussed in Supplementary Information **S3**.

We then select a fixed number of *seed* experiments with the best *p*-values from the *t*-tests. The algorithm builds experiment lists iteratively starting from these seed experiments. For a given list, at each iteration, an experiment is selected at random among those not already contained in the list and this experiment is tentatively added to the existing list. As before, a *t*-test is then performed to check whether this expanded list of experiments exhibits a distribution of correlations between gene pairs where both genes belong to the functional category of interest (A in Figure 3) which is greater than correlation between gene pairs where only one gene belongs to the functional category of interest (B in Figure 3). If the *p*-value is smaller than a pre-defined threshold, the experiment is permanently added to the list; otherwise it is removed. This iterative procedure terminates when all experiments have been considered for every seed experiment for every list. Once the lists have all been created, the list with the overall final best *p*-value is kept as the optimal list of experiments that the algorithm returns. Finally, it should be pointed out that a *t*-test requires that the values being tested be independent samples from a Gaussian distribution. In our case, the values being tested are the pair-wise correlations in a set of genes. Unfortunately, such correlations are neither independent nor Gaussian. Thus, the *p*-values computed by our algorithm are not guaranteed to be accurate. Nevertheless, they are still very useful for choosing experiments. This issue is more fully discussed in the Discussion section.

Although this algorithm cannot guarantee that the selected set of experiments is optimal, in practice we found that this heuristic selected sets of experiments with high discriminatory ability while providing computational tractability. Indicating with *n* the number of experiments in the dataset and with K the number of seed experiments, the number of *t*-tests our algorithm needs to consider at most is given by:


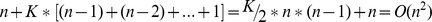


This quadratic complexity allowed us to complete one run of the algorithm for any of the experiments presented here in a few minutes on a regular desktop machine.

The algorithm has only two parameters: the significance level of the *t*-tests (denoted by L in the pseudocode) and the number of seed experiments (K). When testing our algorithm we set the significance level to the standard value of 0.05. Importantly, we found that our algorithm is quite insensitive to the number of seed experiments – in the experiment presented in the sequel, in which we tested the procedure on different species and different sets of microarray experiments, a value of K = 25±15 gave similar results.

Compared to large collections of microarrays, smaller subsets of experiments may lead to higher correlation values purely because of the shorter length of the vectors. In all our analyses we account for this bias by filtering the correlation by a *p*-value threshold. This ensured that only statistically significant correlations are considered.

We tested our algorithm on publicly available microarray data collections. Here we present results obtained using 44 individual experiments in *Arabidopsis thaliana* from the NASCAarray collection [9] and 31 individual experiments in *Saccharomyces cerevisiae* from the M3D collection [10]. A full list and details of the microarray experiments can be found in Supplementary Information **S4**. Our experiments on both yeast and Arabidopsis prove that our procedure is also species-independent. To prove that our selection procedure is independent of the functional classification system adopted, we applied our algorithm for selecting experiments relevant for GO Biological Process terms and MIPS FunCat terms.

In the following sections, we will prove the effectiveness of our procedure by showing that the selected sets of experiments: result in higher correlations between genes in the same functional category; improve the performance of GBA-based gene function prediction; provide a discriminatory ability for a given functional term which increases with the term specificity; lead to a better reconstruction of gene regulatory networks. Furthermore, in the discussion we shall highlight how the selected experiments can also be explained in terms of the literature.

### (a) Selected experiments improve overall correlation between genes in the same functional category

**Figure 4:**
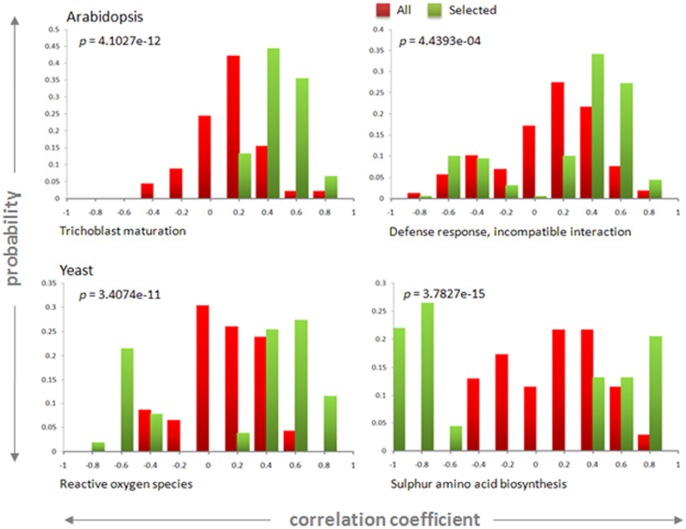
Experiments selected by the algorithm improves correlations between gene pairs. Distribution of correlation coefficients for genes in different GO BP terms when using the experiments selected by our algorithm (green) and when using all the experiments (red). Each quadrant shows a different GO BP term, two terms are for Arabidopsis and two for yeast. The *p*-values of the *t*-test between each pair of distributions indicate that the distributions of correlations for the selected experiments are significantly greater than the corresponding distributions for all experiments.

As discussed earlier, for effective gene expression-based functional analyses, it is essential that genes belonging to the same functional category exhibit high correlation. However, we observed that this is not necessarily true when large microarray collections are used for calculating the correlation (Fig. 1). The experiments selected by our algorithm uncover significantly higher correlation. For genes which belong to the same functional category, we compared the distribution of correlation coefficients obtained using the experiments selected by the algorithm with the distribution obtained using all the experiments in the collection. Figure 4 shows representative histograms of the distributions for both Arabidopsis and yeast GO terms. As expected, the correlation distribution obtained from using all experiments is populated with low correlations with only a greatly reduced population with higher correlation values. However, when experiments selected by the algorithm are used, the distribution is enriched with higher positive and negative correlations. We performed a *t*-test between the distributions of the absolute values of the correlations in order to check whether the distribution obtained using selected experiments is greater than when all experiments are used. The low *t*-test *p*-values (Figure 4) confirm that the distribution obtained with selected experiments is significantly higher than when all experiments are used.

### (b) Quantifying the effectiveness of the selected experiments at improving the correlation in a functional category

In order to evaluate the effectiveness of the selected experiments, we formulated a classification problem where pairs of genes are classified into two classes: the first class contains the pairs were both genes belong to the category of interest; the second class contains those pairs in which one gene belongs to the category of interest while the other one belongs to the background set. This classification is performed using the Pearson correlation between the genes in the pair as the only feature. This allowed us to compare the effect of using the selected experiments vs. using all experiments by comparing the performance of the classifiers – since the classifiers use only correlation to distinguish between the two types of gene pairs, comparing them allows us to assess the quality of the correlations.

Receiver Operating Characteristic (ROC) curves are widely used in the machine learning literature for comparing classifiers. They are the plot of the True Positive Rate (TPR) against the False Positive Rate (FPR) for different values of the classifier decision threshold. The greater the area under the ROC curve (AUC), the better is the performance of the classifier.

**Figure 5:**
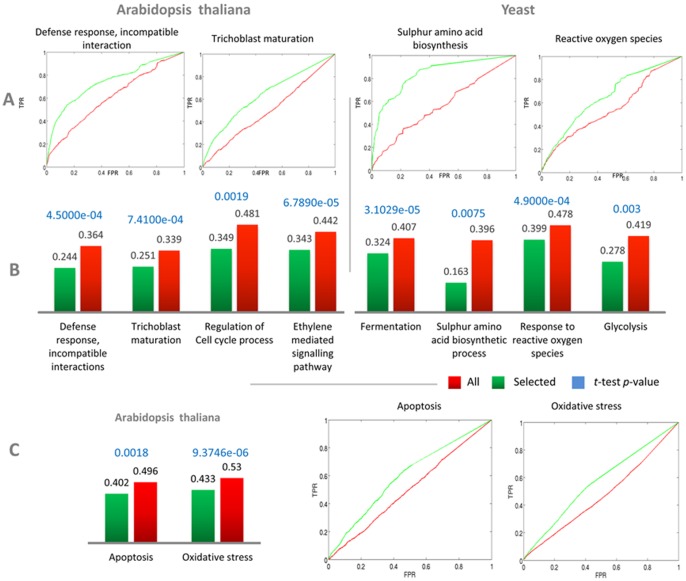
ROC curve analyses quantify the effectiveness of the experiment selection algorithm. ROC curve analysis for evaluating the effectiveness of the selected experiments at improving the correlation between genes in the same functional category (further examples for both the GO and MIPS functional categorizations are presented in Supplementary Information **S6**). (**A**) Average ROC curves from 10-fold cross validation obtained using all experiments (red) and the experiments selected by our algorithm (green) for four different GO functional categories, two from Arabidopsis and two from yeast. (**B**) Averaged (1-AUC) scores from ten-fold cross validations – the lower the value of (1-AUC), the better the performance of the classifier. (**C**) Average (1-AUC) and average ROC curves for two MIPS FunCat terms for Arabidopsis. *p* values for the *t*-test between the ten (1-AUC) values from the ten-fold cross-validation obtained using the selected experiments and those obtained using all experiments are reported in blue for both (B) and (C).

For calculating the ROC curves, gene pairs in which both genes belong to the functional category of interest were considered the Positive Set; and gene pairs, in which only one gene belongs to the functional category of interest, were considered the Negative Set. To evaluate the performance of the selected experiments, we performed a 10-fold cross-validation: genes were first randomly divided into 10 parts and at each round, 9 parts were used for training and one for testing. At each round, our algorithm was used to select the experiments using only the training set and these were then used for calculating the ROC curves for the testing set. Figure 5A shows the average ROC curves for four different GO functional categories, two from Arabidopsis and two from yeast (the procedure for averaging ROC curves can be found in [11]). We can see that the ROC curves for selected experiments (shown in green) have a greater AUC compared to all experiments (shown in red). Following common practice, we also present the average (1-AUC) for both selected and non-selected datasets over the 10-folds (Fig. 4B). The average ROC curves and their corresponding (1-AUC) for further twelve examples of different GO functional categories can be found in Supplementary Information **S5**.

We can see that the average (1-AUC) is remarkably lower for the selected set of experiments. Moreover, we performed a *t*-test between the ten (1-AUC) values from the 10-fold cross-validation obtained using the selected experiments and those obtained using all experiments – *p* values are also reported in Figure 5. This proves the clear difference in performance between classifiers that use all experiments and classifiers that use experiments selected by the algorithm and, consequently, the improved quality of the correlations obtained when selecting experiments.

The superior performance of the selected set of experiments was observed for both Arabidopsis and yeast GO Biological Process terms (Fig. 5A, Fig. 5B). Importantly, we were able to show that this effect is true also for MIPS FunCat terms, thus indicating that our procedure is effective independently of the functional categorization adopted. Figure 5C shows the average ROC curves and their corresponding (1-AUC) together with their *p* values for 2 MIPS functional categories – further twelve examples of MIPS categories can be found in Supplementary Information **S5**. In general we found that for MIPS FunCat terms the difference in performance between the selected experiments and all experiments was smaller compared to GO Biological Process terms. This could be due to the broad functional classification found in MIPS FunCat when compared to GO (we discuss the relation between specificity of annotation and performance of the selected set of experiments in the following section (d)).

### (c) Selected experiments improve GBA-based gene function prediction

A central goal of GBA-based analysis of transcriptomics data is to predict gene function. Therefore an important test of the efficacy of our method is to check whether the correlations obtained by the selected experiments are a better feature for predicting gene function than the correlations obtained using the entire set of experiments.

We framed this problem as a classification problem between two classes of genes: those in the category of interest and those in the background. This classification is performed using very simple GBA-inspired classifiers that use only the Pearson correlation between the genes. The simplest possible classifier of this kind is one that classifies a gene using the sum of the correlations between that gene and the genes in the training set that belong to the category of interest: if this sum is above a certain threshold, it classifies the gene as belonging to the category of interest; otherwise it assigns it to the background.

**Figure 6:**
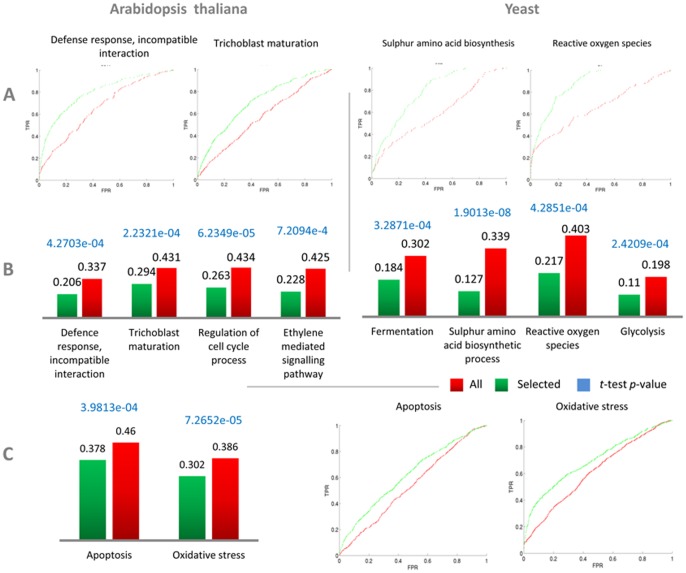
Experiments selected by the algorithm improve GBA-based function prediction. ROC curve analysis for evaluating the effectiveness of the selected experiments at improving GBA-based gene function prediction. The functional categories shown here are the same as in Figure 5, while more examples for both the GO and MIPS functional categorizations are presented in Supplementary Information **S7**. (**A**) Average ROC curves from 10-fold cross validation obtained using all experiments (red) and the experiments selected by our algorithm (green) for four different GO functional categories, two from Arabidopsis and two from yeast. (**B**) Averaged (1-AUC) scores from 10-fold cross validation – the lower the value of (1-AUC), the better the performance of the classifier. (**C**) Average (1-AUC) and average ROC curves for two MIPS FunCat terms for Arabidopsis. *p*-values for the *t* test between the ten (1-AUC) values from the ten-fold cross-validation obtained using the selected experiments and those obtained using all experiments are also reported in blue for both (B) and (C).

As before, to evaluate the performance of the classifiers we performed a 10-fold cross-validation and calculated the average ROC curves. Our aim is to compare the performance of classifiers that employ correlations from the selected experiments with the performance of classifiers that employ correlations from the entire set of experiments. Figure 6A shows the average ROC curves for four different GO functional categories, two from Arabidopsis and two from yeast – the categories are the same ones that we used in Fig. 5. We can see that the ROC curves for selected experiments (shown in green) have a greater AUC compared to all experiments (shown in red). The (1-AUC) for both selected and non-selected datasets over the 10-folds is shown in Figure 6B, together with the *p* values obtained by the *t*-test of the (1-AUC) values obtained in the 10-fold cross-validation. As before, this proves the clear difference in performance between classifiers that use all experiments and classifiers that use experiments selected by the algorithm and, consequently, the improved quality of the correlations obtained when selecting experiments. The average ROC curves and their corresponding (1-AUC) for further twelve examples of different GO functional categories can be found in Supplementary Information **S6**. Results for yeast also show the same effect (Fig. 6A, Fig. 6B).

We also repeated our experiments for MIPS FunCat terms obtaining consistent results (see figure 6C – further twelve examples of MIPS categories can be found in Supplementary Information **S7**). Again, this indicates that our experiment selection procedure is effective at improving GBA-based gene function prediction independently of the functional categorization adopted.

### (d) The effectiveness of the selected experiments increases with annotation specificity

Another way to prove the effectiveness of our experiment selection procedure is to show that the performance of the selected experiments for the classification task outlined in section (b), increases as the functional category becomes more specific. This is based on the fact that the overall correlation among genes in a functional category is expected to increase as the category becomes more specific. Consequently, a set of relevant experiments should be more effective in differentiating genes belonging to the functional category of interest from all other functional categories. To evaluate this, we measured the effectiveness of the selected experiments at every level of specificity of annotation starting from a leaf node up to the root node.

**Figure 7:**
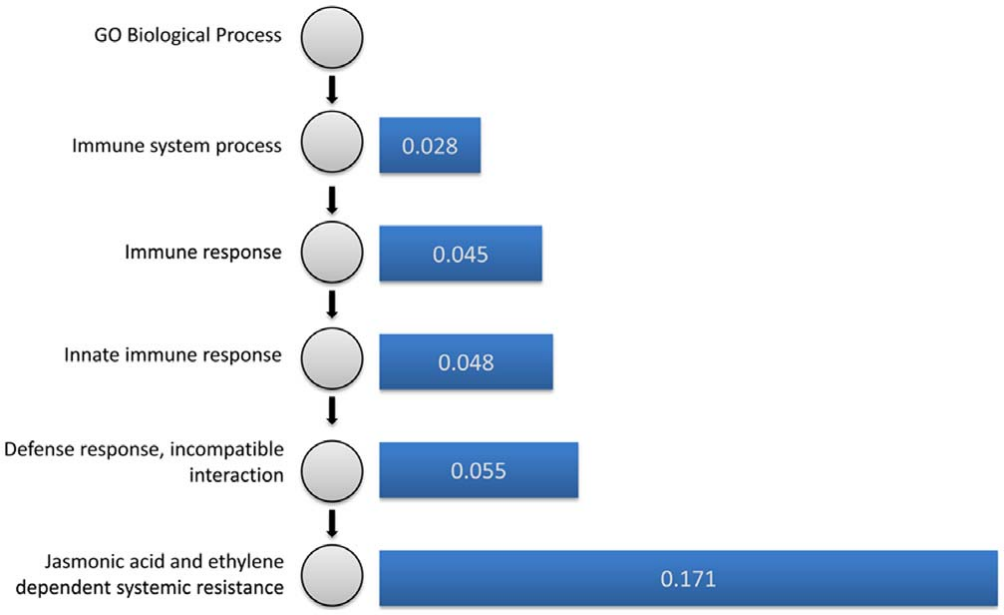
The effectiveness of selected experiments improves with annotation specificity. Difference between the average (1-AUC) of the 10-fold cross-validations, obtained for the selected set and for all experiments, for every term from the leaf node “GO:0009861: Jasmonic acid and ethylene dependent systemic resistance” up to the root. The greater the difference, the better the performance of the selected set.

For the GO BP term GO:0009861 “Jasmonic acid and ethylene dependent systemic resistance”, we ran our algorithm for terms found at each level of the tree leading up to the root term. The effectiveness of the selected experiments was evaluated using the classification problem framework outlined in section (b). The performance of the classifier was evaluated by plotting ROC curves and the average (1-AUC) score in 10-fold cross-validation was recorded. Here, we expect that if the performance of the selected experiments were the same as using all experiments then the difference in their average (1-AUC) scores would be zero. This difference for every term in the hierarchy from GO:0009861 up to the root node is shown in Figure 7. From the figure, it is clear that the effectiveness of the selected experiments is dependent on the specificity of the functional annotation.

### (e) Selecting relevant experiments: Implications on Pathway reconstruction

An important application of the GBA principle is the elucidation of putative members of biological pathways. Identifying experiments relevant to the pathway of interest can be crucial for pathway reconstruction methods where the objective is to identify potential members of a pathway. The same reasoning we applied earlier for selecting experiments relevant to specific functional categories can also be applied for selecting experiments relevant to given biological pathways. In this case, the background set is constituted by all the genes belonging to pathways different from the pathway of interest, and the set of relevant experiments are the ones which best discriminate genes in the pathway of interest from the background. The results we present here show that the selected experiments can uncover greater correlation among genes belonging to the biological pathway and that this correlation is a better predictor of the membership of a gene in the pathway of interest.

**Figure 8:**
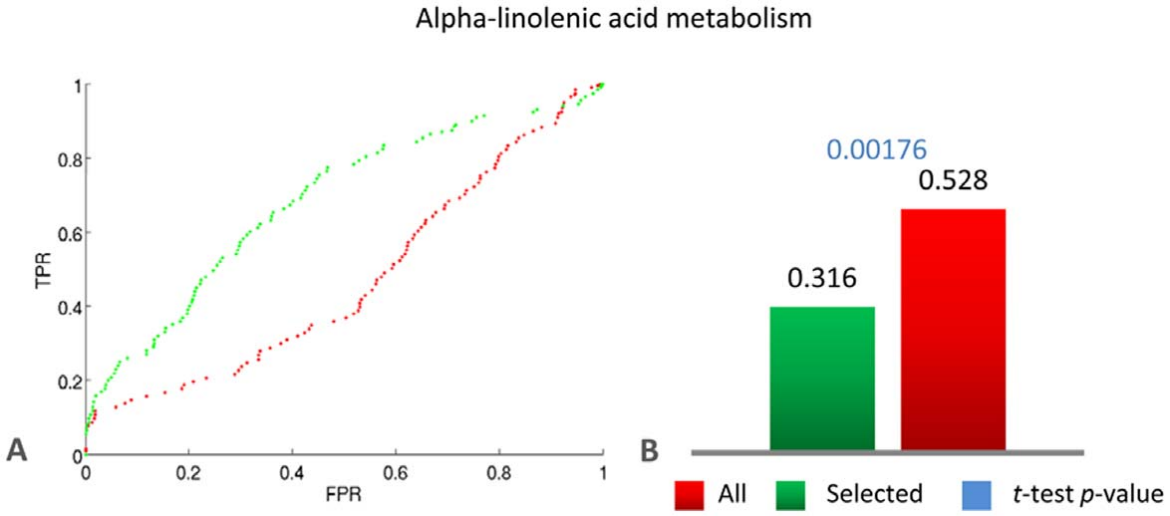
The experiment selection algorithm is effective on gene groups based on KEGG. ROC curve analysis for genes in the “Alpha linolenic acid metabolic pathway” (KEGG ID: ath00592) from the KEGG Pathway Database. (**A**) Average ROC curves from the ten-fold cross-validation for the GBA-based classifier for predicting genes belonging to “Alpha-linolenic acid metabolism” pathway. (**B**) Average (1-AUC) scores from 10-fold cross validation. The *p*-value for the *t*-test between the ten (1-AUC) values from the ten-fold cross-validation obtained using the selected experiments and those obtained using all experiments is also reported in blue.

To begin with, we obtained the “Alpha linolenic acid metabolic pathway” (KEGG ID: ath00592) from the KEGG Pathway Database [12]. Alpha linolenic acid is a precursor of a class of fatty acid derived regulators called the Jasmonates. The biosynthetic derivative of alpha linolenic acid Jasmonic acid (JA) is known to be an important mediator of defence response and other stress related signalling in plants [13], [14]. The KEGG annotation of the pathway in *A.thaliana* consists of 30 genes of which 26 were found in our microarray collection. To demonstrate that the correlation obtained from the selected set of experiments is a better predictor of pathway membership, we framed this as a classification problem similar to the one presented in Section (c). We applied the same GBA-based classifier presented in section (c) to classify genes as either members of the pathway of interest or of the background. As before, the performance of the classifier was evaluated by 10-fold cross-validation and average ROC curves were calculated over the ten folds. We compared the performance of the classifier when using correlations from the selected experiments and all experiments in the collection. The average ROC curves (Figure 8) and the average (1-AUC) bar plots (Figure 8) clearly show that the classifier using correlations from the selected set outperforms the classifier using correlations from all experiments in the collection. This result clearly highlights the potential of the experiment selection algorithm in pathway modelling and reconstruction approaches.

## Discussion

In this paper, we discussed the significance of using only relevant transcriptomics datasets in GBA-based functional analyses. The idea of identifying relevant experiments reflects the discussion by Adler et al. [7] who acknowledged the pitfalls of using large microarray collections in co-expression analyses and suggested manually selecting relevant datasets based on literature knowledge. However, manual selection is progressively becoming unfeasible with the ever increasing size of microarray databases. Furthermore, the relevance of an experiment for a certain functional class may not be obvious. We developed an algorithm which is able to identify a set of experiments from a microarray collection that can improve GBA-based analyses and we demonstrated its effectiveness in different ways.

Firstly, we showed that across various functional categories, for the selected set, the histograms of correlation coefficients show enrichment in larger (in absolute value) correlation coefficients (Fig. 4). Secondly, this enrichment was also shown by comparing classifiers in a machine learning framework (Fig. 5). Thirdly, we showed that the selected experiments improve GBA-based gene function prediction (Fig. 6). Additionally, we observed that the performance of the selected set varied with the specificity of the functional annotation (Fig. 7): as broader process annotations contain several smaller more specific processes, the overall correlation between the genes would be relatively lower than in specific processes. As a result, it is harder to differentiate the process from the background.

Moreover, our results on Arabidopsis and yeast show that the algorithm performs in a consistent way, independently of the type of organism. We also see that the selection performance is independent of the functional classification system (results using GO or MIPSFunCat were comparable).

As mentioned in the Results section, the conditions for use of the *t*-test are not strictly met as they would require the correlations to be independent and Gaussian. Neither condition is met by the correlations in a set of genes. Clearly they are not Gaussian, since Gaussian data is unbounded, while correlations are bounded between −1 and 1. In addition, they are not independent. For example, if g_1_, g_2_ and g_3_ are three genes, then, in general, corr(g_1_, g_3_) is not independent of corr(g_1_, g_2_) and corr(g_2_, g_3_) – for instance, if corr(g_1_, g_2_) and corr(g_2_, g_3_) are both high, then corr(g_1_, g_3_) must also be reasonably high (here, corr(g_i_, g_j_) denotes the correlation between genes g_i_ and g_j_).

Thus, the use of *t*-tests here is not guaranteed to give accurate *p*-values. However, our motivation for using the *t*-test is not to compute accurate *p*-values, but to compensate for the different number of microarrays in the different sets of experiments, something for which the *t*-test seems well-suited. In particular, since the computed *p*-values are used only to rank experiments, precise *p*-values are unimportant, as long as their *relative* values are approximately correct. Importantly, our results show that the *t*-test is a promising heuristic. Finally, we point out that a possible future refinement of this work is to apply a Fisher transformation to the correlation data to make it approximately Gaussian before applying the *t*-tests. Although this would not eliminate the dependences between the correlations, it might result in a more powerful test.

Observing the biological background of the experiments selected as relevant by the algorithm we note that most of the selections are also in agreement with literature knowledge. For example, in the results obtained for Arabidopsis, the selection of experiments for GO:0009873 “Ethylene mediated signalling pathway” such as osmotic stress time series, salt stress time series and oxidative stress time series experiments seem relevant as ethylene is a well-studied mediator of osmotic stress- and salt stress-related responses [15]. Also, ethylene along with hormones such as abscisic acid (ABA) has been shown to control many of the drought-related responses [16]. For growth-related terms such as GO:0010564 “Regulation of cell cycle process” and GO:0048764 “Trichoblast maturation”, growth-related experiments such as the Weigel developmental stages experiments were selected. For the GO term GO:0010053 “Root epidermal cell differentiation”, experiments related to ABA treatment and ethylene treatment were selected. These selections are reasonable as studies such as [17] have demonstrated the role of ABA, along with hormones such as ethylene in regulating epidermal cell-specific gene expression in *Arabidopsis thaliana* roots. Similarly, yeast experiment selections also generally reflected the functional backgrounds of the functional category of interest. Examples of GO BP terms and the corresponding sets of experiments selected by our algorithm are provided in Supplementary Information **S7**.

It is worth highlighting that a minority of experiments selected by the algorithm seemed to be unrelated to the GO terms of interest. We found this reasonable as the Biological Process of interest could also be activated in experiments originally designed to study a seemingly unrelated phenomena; in other words, this could be due our current limited biological understanding about these processes. Viewing the experiment selection procedure as a classification problem also provides an insight into the role of the seemingly irrelevant experiments in the selected set. In fact, it is possible that such experiments may not be good “descriptors” of the functional category of interest. However, they may be effective discriminators of the functional category of interest from the background. An analogous example would be a classification problem, where spheres have to be identified from a collection of objects containing cubes, pyramids and spheres. Several features exist that can effectively describe a sphere. However, in addition to these features, a feature such as the lack of sharp corners between the faces in the object can be very effective for discriminating the sphere from all the other objects in the collection. In this example, it is interesting to note that although the angle between the edges would not be relevant to describe a sphere, it is nevertheless effective for discriminating the sphere from all the other objects in the collection. Similarly, the set of experiments selected as relevant to a functional category of interest may contain experiments which do describe that functional category, but nonetheless may be very relevant in a GBA-based functional analysis. We note that it would not be possible to identify such experiments based on literature knowledge alone.

The algorithm is highly scalable and can be efficiently deployed to select experiments from large microarray collections. The execution time of the algorithm can be further reduced by sampling only a few genes from each functional category used in the background set. Although in the results presented in this paper we always used the full set of genes in the background set, we find that, in general, using this sampling technique provides good results while greatly reducing the computational time (data not shown). One of the important applications of our experiment selection algorithm is the selection of relevant datasets for specific biochemical pathways. We see that with the selected set of experiments, the members of the pathway show stronger correlation among themselves compared to the correlation in the background set and the correlation with the background genes. Thus the selected set increases the likelihood of detecting true members of the pathway of interest.

In conclusion, we believe that our semi-supervised experiment selection method can have a wide reaching impact for gene network construction, gene function prediction and biochemical pathway modelling.

## Materials and Methods

### Data Preparation

For *Arabidopsis thaliana*, our microarray data collection consisted of 756 Affymetrix ATH1-501 22 K arrays from 44 experiments. The microarrays were sourced from NASCARRAYS [9] Pathogen Series, Developmental Series, Stress series and Chemical and Hormone treatment series. Raw data was downloaded, pre-processed and normalized by MAS 5.0 using R Bioconductor packages [18]. All the data used were from experiments based on wild-type plants only. Experiments conducted on multiple organs such as roots and shoots were considered as separate experiments. For yeast, the microarray collection consisted of 537 Affymetrix microarrays from 31 individual experiments. The data was downloaded from the Many Microbes Database [10] and consisted of a mix of wild-type and mutant based experiments under various stresses, growth, chemical and hormone treatments.

Throughout our analysis, only GO Biological Process annotations with non-electronic evidence codes were considered. The background set comprised of genes which belong to GO terms other than the term of interest and its children. In order to afford sufficient number of genes for a statistically significant *t*-test and cross-validation, only terms with at least 25 genes were chosen as the category of interest. Similarly, for MIPS FunCat, the term of interest included all its children in the tree; the remaining terms were considered as the background. For the pathway analysis, pathways and genes annotated to the pathways were obtained from KEGG [12].

The *p*-values for the correlation coefficients were calculated using the *corrcoef* function of MATLAB. *corrcoef* transforms the correlation to create a *t*-statistic having *n*−2 degrees of freedom, where *n* is the number of rows in the data. The confidence bounds are based on an asymptotic normal distribution of 0.5*log((1+R)/(1−R)), with an approximate variance equal to 1/(*n*−3), where R is the sample correlation.

## Supporting Information

Supplementary Information S1 Poor correlation can be an experimental artefact.
(DOCX)

Supplementary Information S2 Poor correlation can rapidly dilute the average correlation.
(DOCX)

Supplementary Information S3 Discussion on the use of the correlations between genes in the background.
(DOCX)

Supplementary Information S4 List of experiments in the microarray collection.
(DOCX)

Supplementary Information S5 Evaluating the effectiveness of the selected experiments – additional results for GO and MIPS.
(DOCX)

Supplementary Information S6 Selected experiments improve gene function prediction – additional results for GO and MIPS.
(DOCX)

Supplementary Information S7 Examples of experiments selected by the algorithm.
(DOCX)
