# Supplementary material for: Computational Selection of Transcriptomics Experiments Improves Guilt-by-Association Analyses
Source: PLoS One. 2012 Aug 7;7(8):e39681. doi: 10.1371/journal.pone.0039681 (PMC3413680; doi:10.1371/journal.pone.0039681)
Supplement: Supplementary Information S1 — Poor correlation can be an experimental artefact. (DOCX) [file pone.0039681.s001.docx]

**S1. Poor correlation can be an experimental artefact**

We hypothesized that one of the causes of poor correlations could be experimental noise. To understand this, we considered an artificial toy example in which two genes X and Y belong to the same functional category. We assumed that their expression is perturbed by a treatment at time zero and that their expression profiles are measured over 60 time points (Figure S1A). The blue and red curves show the “ideal” noiseless profiles when the treatment is such that the two genes are activated. Their correlation is very high (r = 0.99). However, noise is also present in real measurements, and we would obtain the orange and the blue points labelled as “gene X + noise” and “gene Y + noise”, respectively. Despite the presence of noise, the correlation between these genes is still very high (r = 0.98). We compared this to a scenario where gene X and gene Y are not perturbed by the treatment. In the “ideal” noiseless case, their level of expression would not change, and their correlation would still be very high. However, in real measurements, in the absence of any signal and in the presence of noise, we found the correlation between the genes to be very low (r = -0.11 in the example shown in Figure S1B).


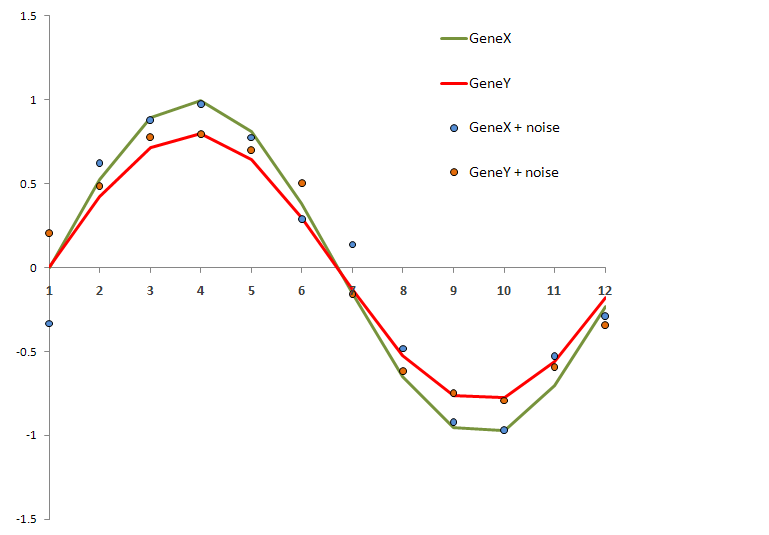


**Figure S1A**


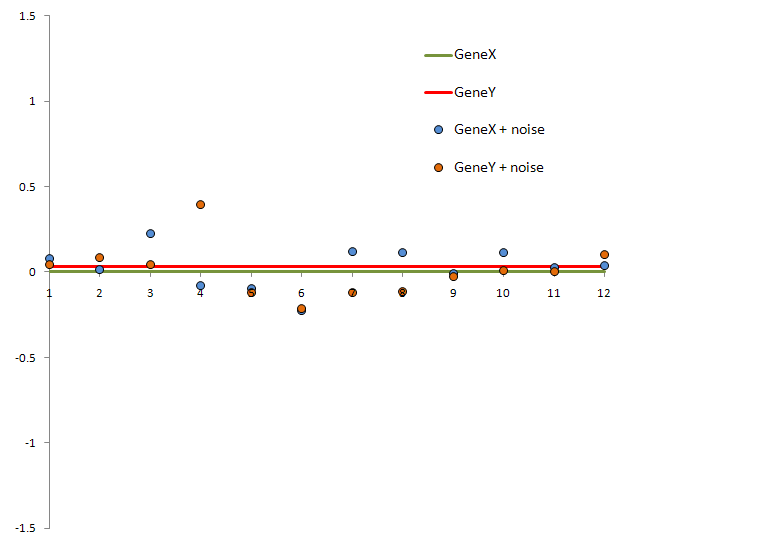


**Figure S1B**
