# Supplementary material for: Computational Selection of Transcriptomics Experiments Improves Guilt-by-Association Analyses
Source: PLoS One. 2012 Aug 7;7(8):e39681. doi: 10.1371/journal.pone.0039681 (PMC3413680; doi:10.1371/journal.pone.0039681)
Supplement: Supplementary Information S2 — Poor correlation can rapidly dilute the average correlation. (DOCX) [file pone.0039681.s002.docx]

**S2. Poor correlation can rapidly dilute the average correlation**

Instances of poor correlation can have a profound influence upon GBA-based analyses as they can dilute the overall correlation. Here we illustrate this through an artificial example by constructing a dataset containing 1000 points (representing 1000 genes), each with 1000 dimensions (representing 1000 experiments, each consisting of one microarray). The first 200 dimensions were constructed such that the points had high correlation (to mimic highly correlating expression profiles, or associated genes). The subsequent 800 dimensions were generated at random to represent gene expression profiles in noisy, poorly correlating, biologically diverse conditions.

**Figure S2**

Figure S2 shows the plot of the average correlation between genes as the number of dimensions increases. For the first 200 dimensions, as expected, the average correlation is very high. However, with the addition of the noisy experiments, the average correlation between genes drops rapidly. Therefore, since correlation values dilute quickly, it is crucial to include in the analysis only those conditions where the genes of interest might be highly correlated.
