# Supplementary material for: Computational Selection of Transcriptomics Experiments Improves Guilt-by-Association Analyses
Source: PLoS One. 2012 Aug 7;7(8):e39681. doi: 10.1371/journal.pone.0039681 (PMC3413680; doi:10.1371/journal.pone.0039681)
Supplement: Supplementary Information S3 — Discussion on the use of the correlations between genes in the background. (DOCX) [file pone.0039681.s003.docx]

**S3. Discussion on the use of the correlations between genes in the background**

Typically, the aim of a GBA based analysis is to identify whether a gene belongs to the functional category of interest. Here, an experiment can be considered relevant to the analysis if it can recognize genes which belong to the functional category of interest based on a selected feature. In our case, we use correlation between genes as the feature.

.


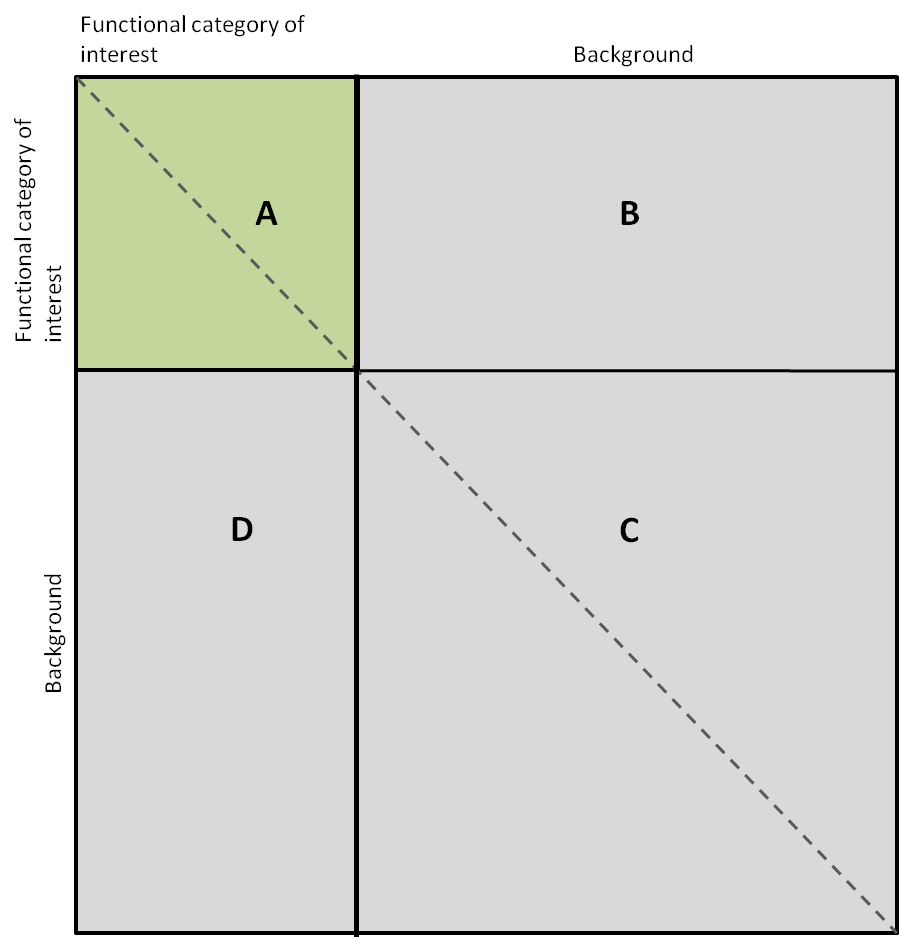


**Figure S3**

In figure S3, we present a pictorial representation of the correlations between genes belonging to the functional category and the background. The correlation for each gene pair is placed in a specific sector of the matrix, according to whether the genes in the pair belong to the category of interest or to the background. We shall now use this figure to explain which sets of correlations are used in our procedure.

Our algorithm performs *t*-tests between correlations in sector A (correlations between genes in the functional category of interest) and correlations in sector B (correlations between genes in the functional category of interest and the background set). Note that elements on the main diagonal of A are equal to one, and that A is symmetric (as indicated by the dashed line). Therefore, in our implementation, we used only the upper triangular part of A. In the same way, we do not use sector D, as it is equal to the transpose of B.

We point out that in our algorithm we do not consider the correlation between pairs of genes which are both in the background set (sector C). This is because the aim of GBA-based functional analyses is to identify genes that are putative members of the functional category of interest based on correlation between genes as the feature. A gene is classified into the functional category based on its correlation with genes that are known members of the functional category. It is important to note that a gene cannot be classified into the functional category of interest if there is no association with any known members of the functional category of interest. That is, any correlation between gene-pairs where neither gene belongs to the functional category of interest is irrelevant to the classification problem. In other words, we assume that genes which belong to the same functional category are likely to show greater correlation compared to their correlation with genes belonging to other functional categories. Therefore, an experiment can be considered relevant if it accentuates the difference between these two correlation distributions, while correlations between genes in the background do not matter for our purpose.

Moreover, in a typical experiment, several biological processes are perturbed and, as a consequence, sector C could be populated by several highly correlated gene pairs. Finally, correlations in sector C could also have high values due to biological events such as cross-talk, perturbation of processes which are functionally related to the functional category of interest or simply noise.
