# Supplementary material for: Computational Selection of Transcriptomics Experiments Improves Guilt-by-Association Analyses
Source: PLoS One. 2012 Aug 7;7(8):e39681. doi: 10.1371/journal.pone.0039681 (PMC3413680; doi:10.1371/journal.pone.0039681)
Supplement: Supplementary Information S4 — List of experiments in the microarray collection. (DOCX) [file pone.0039681.s004.docx]

**S4. List of experiments in the microarray collection**

The table below presents the complete list of the 44 *Arabidopsis thaliana* experiments that we used in our experiments. These experiments were obtained from an original larger set contained in the NASCARRAY database (<http://affymetrix.arabidopsis.info/narrays/experimentbrowse.pl>, accessed on Jan 21^st^, 2010), by removing experiments which were related to mutants and experiments that contained with fewer than 6 data points. This dataset can be downloaded from: http://www.paccanarolab.org/papers/CorrGene/

| **Serial.No** | **NASCARRAY ID** | **Experiment** |
| --- | --- | --- |
| 1 | 152 | Developmental series |
| 2 | 137 | Control (Shoot) |
| 3 | 137 | Control (Root) |
| 4 | 138 | Cold (Shoot) |
| 5 | 138 | Cold (Root) |
| 6 | 139 | Osmotic Stress (Shoot) |
| 7 | 139 | Osmotic Stress (Root) |
| 8 | 140 | Salt stress (Shoot) |
| 9 | 140 | Salt stress (Root) |
| 10 | 141 | Drought stress (Shoot) |
| 11 | 141 | Drought stress (Root) |
| 12 | 142 | Genotoxic stress (Shoot) |
| 13 | 142 | Genotoxic stress (Root) |
| 14 | 143 | Oxidative stress (Shoot) |
| 15 | 143 | Oxidative stress (root) |
| 16 | 144 | UV-B stress (Shoot) |
| 17 | 144 | UV-B stress (Root) |
| 18 | 145 | Wounding Stress (Shoot) |
| 19 | 145 | Wounding Stress (Root) |
| 20 | 146 | Heat Stress (Shoot) |
| 21 | 146 | Heat Stress (root) |
| 22 | 120 | Response to virulent, avirulent bacteria |
| 23 | 122 | Response to bacterial-(LPS, HrpZ, Flg22) and oomycete-(NPP1) derived elicitors |
| 24 | 123 | Response to Phytophthora infestans |
| 25 | 167 | Response to Botrytis cinerea infection |
| 26 | 168 | Pseudomonas half leaf injection |
| 27 | 169 | Response to Erysiphe orontii infection |
| 28 | 172 | ACC time course in wildtype seedlings |
| 29 | 173 | Zeatin time course in wildtype seedlings |
| 30 | 174 | Methyl Jasmonate time course in wildtype |
| 31 | 175 | IAA time course in wildtype seedlings |
| 32 | 176 | ABA time course in wildtype seedlings |
| 33 | 179 | Effect of brassinosteroids in seedlings |
| 34 | 181 | Cytokinin treatment of seedlings |
| 35 | 183 | Effect of ABA during seed imbibition |
| 36 | 184 | Basic hormone treatment of seeds |
| 37 | 185 | Effect of gibberellic acid inhibitors on seedlings |
| 38 | 186 | Effect of auxin inhibitors on seedlings |
| 39 | 187 | Effect of brassinosteroid inhibitors on seedlings |
| 40 | 188 | Effect of ethylene inhibitors on seedlings |
| 41 | 189 | Effect of cycloheximide on seedlings |
| 42 | 190 | Effect of proteasome inhibitor MG13 on seedlings |
| 43 | 191 | Effect of photosynthesis inhibitor PNO8 on seedlings |
| 44 | 192 | Effect of ibuprofen, salicylic acid and daminozide on seedlings |

The yeast microarray data was downloaded from the Many Microbes Database (*S. cerevisiae*). The details of the experiments in the collection can be found at <http://m3d.bu.edu/cgi-bin/web/array/index.pl?section=home> (accessed on October 11^th^, 2010).
