# Supplementary material for: Computational Selection of Transcriptomics Experiments Improves Guilt-by-Association Analyses
Source: PLoS One. 2012 Aug 7;7(8):e39681. doi: 10.1371/journal.pone.0039681 (PMC3413680; doi:10.1371/journal.pone.0039681)
Supplement: Supplementary Information S5 — Evaluating the effectiveness of the selected experiments – additional results for GO and MIPS. (DOCX) [file pone.0039681.s005.docx]

**S5. Evaluating the effectiveness of selected experiments – additional results for GO and MIPS**

In section (b) of RESULTS section, we evaluate the effectiveness of the selected experiments by formulating a classification problem. For a given functional category, we select experiments using our algorithm and evaluate the performance of the classifier with a 10-fold cross-validation routine. The performance of the classifier using selected set is compared to using all experiments in the microarray collection. Below in Figure 4, we present additional examples of GO Biological Process categories, for which we select experiments and measure the performance of the classifier. The average (1-AUC) for the selected experiments is presented in green and in red for all experiments. To show that the average (1-AUC) for the selected experiments is significantly higher than when using all experiments, we performed a *t*-test between the set of (1-AUC) over the ten folds of cross validation in either case i.e. selected and all. The *p*-values from the *t*-test are indicated in blue. Similarly, additional examples for MIPS FunCat terms are shown in Figure 5. In both sets of examples, the Arabidopsis microarray collection was used.


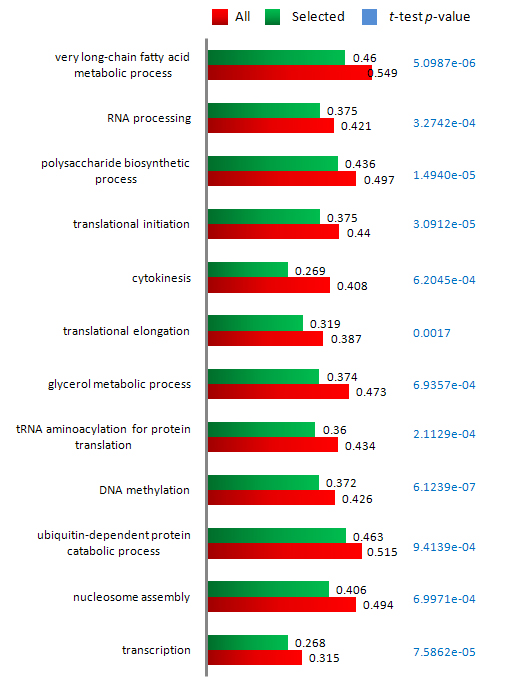


**Figure S4**


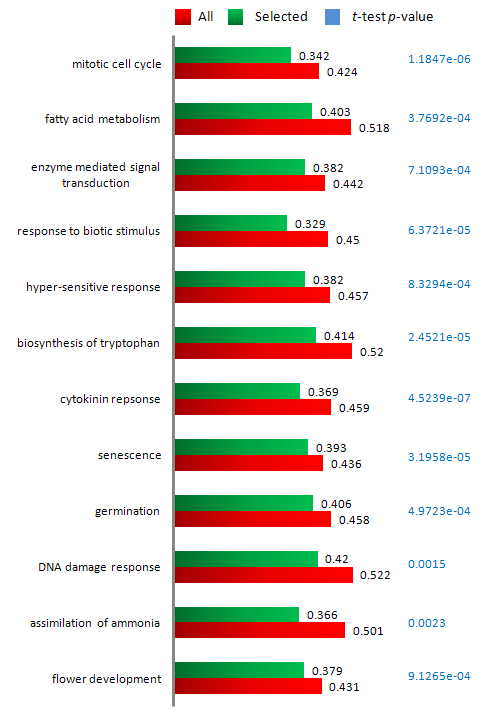


**Figure S5**
