# Supplementary material for: Computational Selection of Transcriptomics Experiments Improves Guilt-by-Association Analyses
Source: PLoS One. 2012 Aug 7;7(8):e39681. doi: 10.1371/journal.pone.0039681 (PMC3413680; doi:10.1371/journal.pone.0039681)
Supplement: Supplementary Information S6 — Selected experiments improve gene function prediction – additional results for GO and MIPS. (DOCX) [file pone.0039681.s006.docx]

**S6. Selected experiments improve GBA-based function prediction – additional results from GO and MIPS**

In the section (c) of the paper, we show that selected experiments improve the performance of GBA-based gene function prediction. We again framed it as a classification problem where a gene had to be classified into either of two classes. The performance of the classifier was measured by plotting average ROC curves over the ten-folds of the cross-validation and the average (1-AUC) was recorded. In Figure 6, for twelve GO Biological Process categories, the average (1-AUC) for the selected set (in green) is compared with the average (1-AUC) obtained by using all experiments (in red). To show that the (1-AUC) obtained for the selected experiments are significantly higher than for all experiments, we performed a *t*-test (*p*-values are shown in blue). Similarly, in Figure 7, results for twelve additional MIPS FunCat examples are shown. In both sets of examples, the Arabidopsis microarray collection was used.


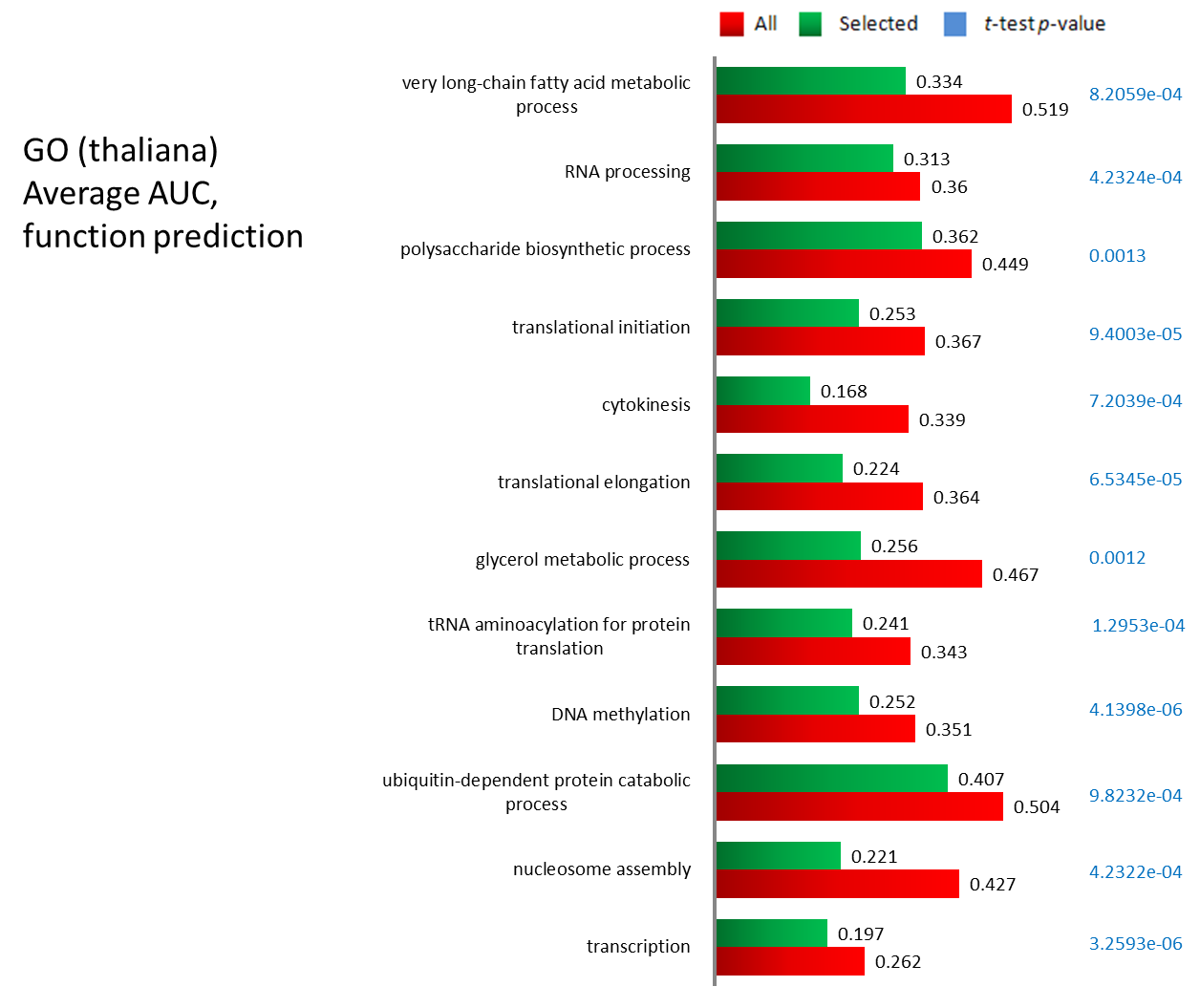


**Figure S6**


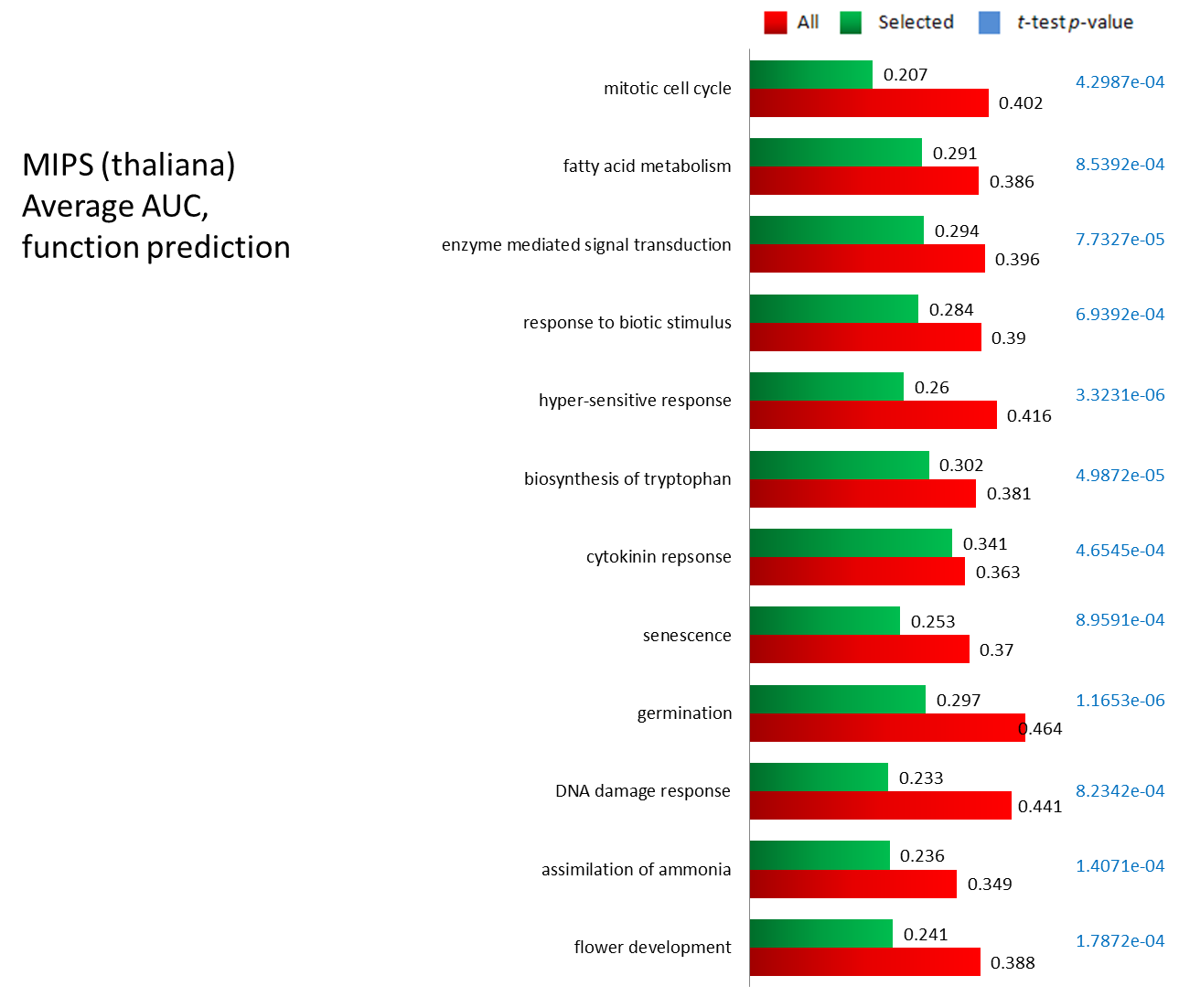


**Figure S7**
