# Supplementary material for: Computational Selection of Transcriptomics Experiments Improves Guilt-by-Association Analyses
Source: PLoS One. 2012 Aug 7;7(8):e39681. doi: 10.1371/journal.pone.0039681 (PMC3413680; doi:10.1371/journal.pone.0039681)
Supplement: Supplementary Information S7 — Examples of experiments selected by the algorithm. (DOCX) [file pone.0039681.s007.docx]

**S7. Examples of experiments selected by the algorithm**

The experiment selection algorithm is aimed for selecting experiments that are functionally relevant to a functional category of interest. Interestingly, we see that the majority of experiments in the selected set have a biological background that is in agreement with literature knowledge. In this section, we present examples of experiment selection for Yeast and Arabidopsis GO Biological Process categories.

| **Yeast** | | | |
| --- | --- | --- | --- |
| **S No** | **GO identifier** | **Description** | **Experiment name** |
| 1 | 12501 | programmed cell death | Chitin treatment |
|  |  |  | Haploinsufficiency experiment |
|  |  |  | Antibiotic treatment |
| 2 | 6031 | chitin biosynthetic process | Antibiotic treatment |
|  |  |  | Aging |
|  |  |  | Xylose in aerobic and anaerobic treatment |
|  |  |  | Antibiotic treatment 2 |
| 3 | 6099 | tricarboxylic acid cycle | Sporulation experiment |
|  |  |  | Metabolic cycle experiment |
|  |  |  | Chemostatic limitation |
|  |  |  | Aging |
|  |  |  | Antibiotic treatment |
|  |  |  | Post drying time series |
|  |  |  | Nitrogen limitation |
|  |  |  | Histone mutant |

| **Arabidopsis thaliana** | | | |
| --- | --- | --- | --- |
| **S No** | **GO identifier** | **Description** | **Experiment name** |
| 1 | 302 | response to reactive oxygen species | Heat stress (root) |
|  |  |  | Cold stress (root) |
|  |  |  | Genotoxic stress (root) |
|  |  |  | Wounding (root) |
|  |  |  | UV-B stress (root) |
| 2 | 9860 | Pollen Tube Growth | Control, no treatment (shoot) |
|  |  |  | Developmental series |
|  |  |  | Cytokinin treatment |
|  |  |  | Salt stress (root) |
|  |  |  | ABA treatment seed imbibition |
|  |  |  | IAA treatment (seedlings) |
|  |  |  | Phytophtora infestans treatment |
|  |  |  | Oxidative stress (shoot) |
|  |  |  | ABA treatment (seedlings) |
|  |  |  | Pseudomonas syringae treatment |
|  |  |  | Zeatin treatment (seedlings) |
|  |  |  | Wounding stress (shoot) |
| 3 | 9867 | jasmonic acid mediated signaling pathway | Psuedomonas syringae treatment |
|  |  |  | Heat stress (shoot) |
|  |  |  | Gibberelic acid treatment (seedlings) |
|  |  |  | PNO8 treatment (seedlings) |
|  |  |  | IAA treatment (seedlings) |
|  |  |  | Ethylene treatment (seedlings) |
|  |  |  | ACC treatment (seedlings) |
|  |  |  | Auxin treatment (seedlings) |
| 4 | 9834 | secondary cell wall biogenesis | Developmental series |
|  |  |  | ABA treatments (seedlings) |
|  |  |  | Heat stress (root) |
|  |  |  | Gibberellic acid treatment (seedlings) |
|  |  |  | ABA treatment seed imbibition |
|  |  |  | Brassinosteroid treatment (seedlings) |
|  |  |  | Cytokinin treatment |
|  |  |  | Botrytis cinerea treatment |
|  |  |  | Drought (shoot) |
|  |  |  | Osmotic stress (root) |
|  |  |  | Bacterial elicitor treatment |
